# Distinct Genomic Integration of MLV and SIV Vectors in Primate Hematopoietic Stem and Progenitor Cells

**DOI:** 10.1371/journal.pbio.0020423

**Published:** 2004-11-23

**Authors:** Peiman Hematti, Bum-Kee Hong, Cole Ferguson, Rima Adler, Hideki Hanawa, Stephanie Sellers, Ingeborg E Holt, Craig E Eckfeldt, Yugal Sharma, Manfred Schmidt, Christof von Kalle, Derek A Persons, Eric M Billings, Catherine M Verfaillie, Arthur W Nienhuis, Tyra G Wolfsberg, Cynthia E Dunbar, Boris Calmels

**Affiliations:** **1**Hematology Branch, National Institutes of HealthBethesda, MarylandUnited States of America; **2**Experimental Hematology Division, Department of Hematology/Oncology, St. Jude Children's Research HospitalMemphis, TennesseeUnited States of America; **3**Genome Technology Branch, National Human Genome Research Institute, National Institutes of HealthBethesda, MarylandUnited States of America; **4**Stem Cell Institute, University of MinnesotaMinneapolis, MinnesotaUnited States of America; **5**Bioinformatics Core Facility, National Heart, Lung, and Blood Institute, National Institutes of HealthBethesda, MarylandUnited States of America; **6**Department of Internal Medicine, University of Freiburg, Freiburg, Germany; **7**Division of Experimental Hematology, Children's Hospital Research FoundationCincinnati, OhioUnited States of America

## Abstract

Murine leukemia virus (MLV)-derived vectors are widely used for hematopoietic stem cell (HSC) gene transfer, but lentiviral vectors such as the simian immunodeficiency virus (SIV) may allow higher efficiency transfer and better expression. Recent studies in cell lines have challenged the notion that retroviruses and retroviral vectors integrate randomly into their host genome. Medical applications using these vectors are aimed at HSCs, and thus large-scale comprehensive analysis of MLV and SIV integration in long-term repopulating HSCs is crucial to help develop improved integrating vectors. We studied integration sites in HSCs of rhesus monkeys that had been transplanted 6 mo to 6 y prior with MLV- or SIV-transduced CD34^+^ cells. Unique MLV (491) and SIV (501) insertions were compared to a set of in silico-generated random integration sites. While MLV integrants were located predominantly around transcription start sites, SIV integrants strongly favored transcription units and gene-dense regions of the genome. These integration patterns suggest different mechanisms for integration as well as distinct safety implications for MLV versus SIV vectors.

## Abstract 2

A primate model of gene transfer into hematopoietic stem cells demonstrated MLV integration around transcription start sites whereas SIV integrated into gene-dense regions, indicating distinct safety implications for each

## Introduction

Integration of proviral DNA into the host cell genome is an essential step in the life cycle of retroviruses. The process begins after a retrovirus enters the cell and the RNA genome is reverse transcribed into double-stranded DNA. Preintegration complexes (PICs) containing linear proviral DNA associated with several viral and cellular proteins (Bushman 1999) either enter the nucleus of nondividing cells through the nuclear pores (lentiviruses) or gain access to chromosomal DNA after dissolution of the nuclear membrane during mitosis (oncoretroviruses). When the PIC associates with the host chromosome, the virally encoded integrase directs the insertion of the proviral DNA into the cellular chromosomal DNA (Hindmarsh and Leis 1999). The provirus is then stably transmitted to all progeny of transduced cells as an integral element of the host genome. Beyond its importance to the reproduction of the virus itself, this distinctive feature of retroviruses accounts for many of the characteristics associated with retroviral infection, including latency and persistence of infection, insertional mutagenesis, and the usefulness of retroviruses as vectors for gene therapy.

Engineered replication-defective retroviruses were introduced over 20 y ago and rapidly became attractive tools for efficient and stable introduction of genes of interest, in particular into hematopoietic stem cells (HSCs). Retroviral gene therapy targeting HSCs has been aggressively pursued because of its potential to treat many congenital and acquired human diseases. Its therapeutic promise was convincingly demonstrated in children with X-linked severe combined immunodeficiency (SCID-X1) and adenosine deaminase deficiency (Aiuti et al. 2002; Hacein-Bey-Abina et al. 2002). Unfortunately, elation over this success was recently tempered when lymphoproliferative disease developed in two children who received genetically modified CD34^+^ cells for treatment of SCID-X1, in association with proviral activation of the *LMO2* transcription factor gene (Hacein-Bey-Abina et al. 2003). These serious adverse events have galvanized investigators to further assess the potential risks associated with gene therapy protocols utilizing retroviral vectors.

For many years, researchers have been aware that retroviral insertional activation of proto-oncogenes can result in tumors. Administration of replication-competent oncoretroviruses to susceptible mouse strains led to tumor development, the result of a high number of repetitive insertion events in vivo during rapid cell proliferation, with outgrowth of a clone containing one or more proviruses activating growth control genes (Dudley 2003). While the possibility of insertional mutagenesis using replication-defective vectors has been discussed as theoretically possible (Cornetta et al. 1991), such risks have been estimated to be extremely low (Moolten and Cupples 1992) based on the assumption that proviral integration into the genome was random (Coffin et al. 1997).

With the readily accessible human genome sequence, mapping studies of retroviral integration sites in cell lines have uncovered nonrandom integration patterns, when studied using wild-type HIV, HIV-derived, or murine leukemia virus (MLV)-derived vectors (Elleder et al. 2002; Schroder et al. 2002; Laufs et al. 2003; Wu et al. 2003; Mitchell et al. 2004). However, these integration patterns have not been investigated in the most relevant primary cells for hematopoietic gene therapy, namely HSCs. HSC transduction by retroviral vectors and their subsequent vector-genome integration patterns can unequivocally be assessed only by transplanting these cells and analyzing vector-containing cells in multiple lineages in vivo long-term, since stem and progenitor cell activity are defined by functional reconstitution of hematopoiesis in vivo. Interpretation of such studies may be more complex due to the potential for integration-specific impact on engraftment or functional properties of primitive hematopoietic cells in vivo. However, large-scale analysis of retroviral integration sites in a relevant long-term large animal model is critical to fully assess the potential risks associated with proviral insertion in this population of cells, prior to implementing new gene therapy trials. These studies may also provide further insights into mechanisms of integration targeting and the impact of integration events on the behavior of hematopoietic cells.

In order to evaluate the integrating vectors currently being developed for gene therapy applications, we compared the integration patterns of MLV and simian immunodeficiency virus (SIV) vectors. MLV vectors have been utilized for over a decade in clinical trials. However, they have a number of limitations, including inefficient transduction of quiescent cells and difficulty in maintaining stable high-level expression from tissue-specific internal genetic control elements. Thus, lentiviral vectors based on HIV or SIV “backbones” have been pursued and shown to overcome these limitations, and are now moving into clinical trials.

Vector-genome junction sequences were retrieved from mature granulocytes and mononuclear cells (MNCs) from rhesus macaques transplanted 6 mo to 6 y prior with mobilized peripheral blood (PB) CD34^+^ cells transduced with an MLV- (Schmidt et al. 2002) or SIV-derived vector (Hanawa et al. 2004), both containing marker genes with no known impact on proliferation or survival of transduced cells (Wu et al. 1998). This model represents a unique opportunity to analyze retroviral insertion patterns in the engrafted progeny of primitive long-term repopulating cells, without interference from confounding factors such as the impact of transgene expression or an underlying hematopoietic disease.

## Results

### Cloning, Sequencing, and Bioinformatic Analysis of Retroviral Integration Sites

We used a modification of the sensitive linear amplification-mediated (LAM)-PCR method (Schmidt et al. 2002) to retrieve and clone the genomic regions adjacent to proviral integration sites from circulating granulocytes and MNCs sampled in rhesus macaques engrafted stably long-term, between 6 mo and 6 y after transplantation of transduced CD34^+^ for the MLV-transduced animals, and 6–7 mo posttransplantation for the SIV-transduced animals. In our extensive prior analysis of 46 rhesus macaques, genetic marking levels and clonal integration patterns are stable by 3–4 mo posttransplantation, and remain stable for up to 6–7 y (Kiem et al. 2004). This approach uses a frequent-cutting enzyme to generate average genomic fragments of 80 bp, thereby circumventing PCR bias against large fragments, while facilitating amplification and cloning. The average length of all analyzed genomic fragments was 159 bp (median 131 bp, range 30–728 bp).

Owing to the close phylogenetic relationship between human and rhesus macaques, we were able to directly align our sequences with the human genome assembly. We considered a sequence as a genuine retroviral integration site only if it (a) juxtaposed to the vector long terminal repeat (LTR), (b) yielded a unique best hit by BLAT software (University of California, Santa Cruz [UCSC] Genome Browser, http://genome.ucsc.edu), and (c) showed at least 90% identity to the July 2003 human genome assembly (Kent, 2002; Karolchik et al. 2003). After several analyses using different cutoffs, we decided to use a conservative alignment cutoff of 90% in order to include most of the orthologous regions between human and rhesus genomes, while discarding sequences of technically poor quality. This cutoff eliminated 5 to 10% of the retrieved sequences, and we verified that omission of these sequences with less than 90% identity from our analysis did not change the overall distribution of the integration sites (Table S1). Using these selection criteria, we have retrieved and analyzed 992 independent unequivocal retroviral integration sites (*n* = 491 for MLV [Dataset S1], and *n* = 501 for SIV [Dataset S2]).

Of the 992 integration sites analyzed, 232 (23%) were distributed among the four major classes of transposable repetitive elements, and therefore could not be mapped to a unique position in the genome. These insertions accounted for 59 of 491 (12%) and 173 of 501 (34.5%) of the MLV and SIV integration events, respectively. Human transposon-derived repeats encompass at least 45% of our genome and their distribution is highly variable, with density varying from 2% to 98% depending on the location (Lander et al. 2001).

### Integration Targets Transcription Units

**Figure 1:**
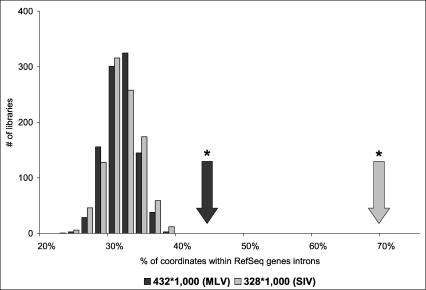
Comparison of MLV and SIV Integration Events Shown are integrations that landed within RefSeq gene introns (arrows) in comparison to in silico-generated integration sites (bars). Black indicates MLV and gray indicates SIV. *p < 0.0001 by a Chi^2^ test.

We correlated the 760 integration sites (432 for MLV and 328 for SIV) that unequivocally mapped to a unique position in the genome to the locations of annotated genes, using the UCSC Genome Browser Reference Sequence (RefSeq) Genes track, which displays the positions of National Center for Biotechnology Information mRNA Reference Sequences (Table 1). We observed that 212 of 432 (49%) of the MLV integrations and 241 of 328 (73%) of the SIV integrations landed between the transcription start and stop point of a RefSeq gene. As a control, we compared the coordinates of two sets of 1,000 in silico-generated random integration sites, each containing 432 or 328 coordinates (760,000 total) with the positions of known genes. Both MLV and SIV insertion patterns were significantly different from the random integration sites (Figure 1), of which only 32 % were within RefSeq genes, a percentage identical to the average estimation of the human genome content (25.5%–37.8%, median 31.6%) (Venter et al. 2001).

**Table 1:**
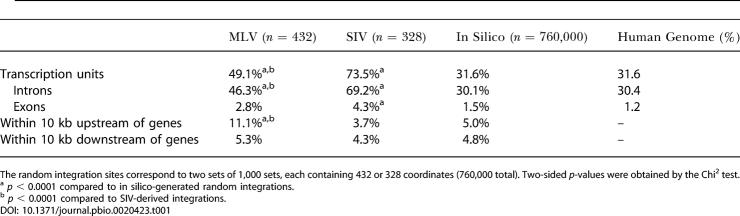
MLV and SIV Integration Sites Distribution (Reported to UCSC RefSeq Genes) Compared to In Silico-Generated Random Integration Sites

The random integration sites correspond to two sets of 1,000 sets, each containing 432 or 328 coordinates (760,000 total). Two-sided *p*-values were obtained by the Chi^2^ test

^a^ *p* < 0.0001 compared to in silico-generated random integrations

^b^ *p* < 0.0001 compared to SIV-derived integrations

10.1371/journal.pbio.0020423.t001

We next examined whether specific regions of the transcription units were more likely sites of integration than others. We analyzed the distribution of the integration events within the transcription unit by dividing the distance of each integration site from the transcription start site by the gene length. The resulting ratio, reported as the total number of integration events in RefSeq genes for each vector, provides the percentage of integrations within ten equal sections of transcription units. While SIV targets the entire transcription unit with no noticeable preference, 42 of 212 (20%) of the MLV integration sites that land within RefSeq genes, as compared 18 of 241 (7%) for SIV, are located within the first one-tenth of the transcription unit, indicating MLV's clear predilection for the 5′ portion of transcription units (*p* = 0.0002).

### MLV Vectors Favor Integration around Transcription Start Sites, and SIV Vectors Integrate Predominantly within Transcription Units

To further explore MLV preferential integration in the vicinity of transcription start sites, we determined the distance to the nearest 5′ and 3′ ends of a RefSeq gene for each integration site. Interestingly, whereas SIV integration events do not favor locations upstream or downstream of transcription units (Table 1), 48 of 432 (11%) of the total MLV integration sites landed within a 10-kb region upstream of a RefSeq gene, as compared to 5% expected with the random integration sets (*p* < 0.0001). The frequency of insertions within 10 kb downstream of the 3′ end is almost identical for the MLV and the in silico-generated random sets (5.3% versus 4.8%).

**Figure 2:**
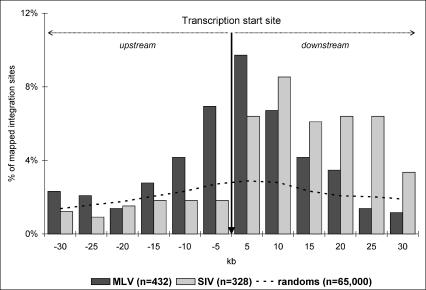
Distribution of MLV and SIV Integration Sites within a 60-kb Window Centered on Transcription Start Sites The vertical arrow points to 0 kb. Each gray bar corresponds to the percentage of SIV integration sites within a 5-kb interval, and black bars correspond to the percentages of MLV integration sites in a 5-kb interval. The distribution of a set of 65,000 in silico-generated random integration sites is represented by the dashed line.

We then looked at the proviral integrations within a 2-kb window on either side of transcription start sites. This survey revealed a strong tendency for MLV vectors to integrate close to transcription start sites, with 46 of 432 (11%) of the total MLV integration events occurring within 2 kb upstream or downstream, as compared to 7 of 328 (2%) for SIV (*p* < 0.0001). We broadened this analysis to a 60-kb window centered on transcription start sites (Figure 2). The overall distribution of the 432 MLV integration events upstream and downstream of transcription start sites is almost identical (20% versus 27%, *p* = 0.02), but their distribution is clearly nonrandom and favors a 10-kb window centered around transcription start sites. This pattern is markedly different from the distribution of SIV sites: Although there is no predilection for integration in the vicinity of transcription start sites, there is a strong preference for integration within transcription units, rather than upstream of them. Of the SIV-derived sites, 122 of 328 (37%) are within 30 kb downstream of the transcription start site, while only 30 of 328 (9%) are within 30 kb upstream (*p* < 0.0001). Taken together, these data show a distinct integration pattern between MLV- and SIV-derived vectors (*p* < 0.00001 using an omnibus contingency Chi^2^ test): While the latter appear to integrate predominantly within transcription units, MLV vectors strongly favor integration within a 10-kb window centered on transcription start sites.

### SIV-Derived Vectors Favor Integration within Gene-Dense Regions of the Genome

**Figure 3:**
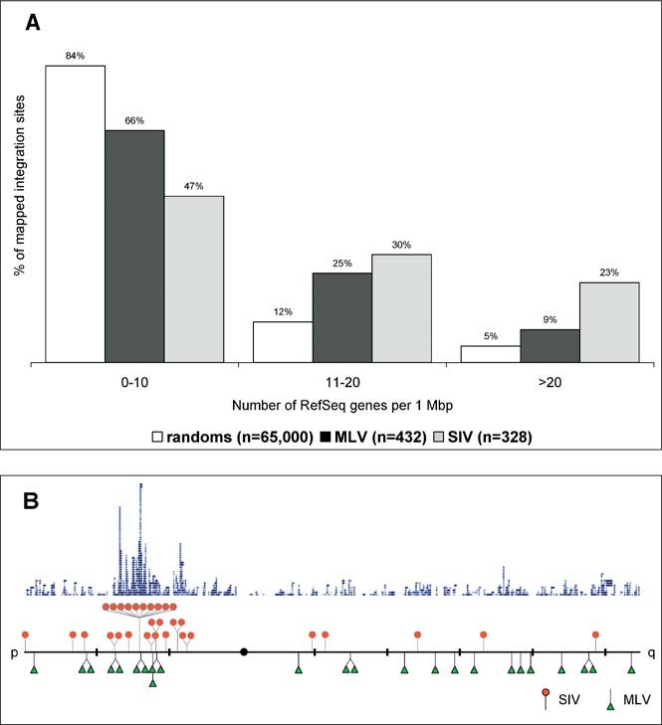
Distribution and Location of Integration Sites Relative to Chromosomal Gene Density (A) Distribution of MLV and SIV integration sites relative to gene density within a 1-Mbp window compared to in silico-generated random integration sites. Each bar corresponds to the percentage of integration sites within the corresponding gene density region. (B) Location of MLV and SIV integration sites and gene density on human Chromosome 6. MLV and SIV integrations were aligned to Chromosome 6 (obtained from the UCSC custom annotation track feature) and shown in relation to RefSeq gene density (blue). 73% of the SIV integration events are within the 20-Mbp unique ridge of Chromosome 6, compared to 29% for MLV. Distance between thick black bars is 20 Mbp; centromere is represented by the black circle.

In order to ask whether the preferential integration of SIV vectors within transcription units might be associated with physical properties of the genome such as gene density, we analyzed the overall distribution of integration sites. The highest density of SIV integration sites per Mbp are on Chromosomes 17, 19, and 22 (0.50, 0.25, and 0.27 respectively), the three most gene-dense chromosomes, with 15, 23, and 17 genes per Mbp, respectively (Venter et al. 2001). Since each chromosome is a patchwork of domains with varying gene density, we determined the number of RefSeq genes within 1 Mbp of every integration site's LTR coordinate (Figure 3A). While most (84%) of the random integration sites tended to be within regions of average gene density (0–10 genes per Mbp), MLV displayed a strong tendency to integrate within more gene-dense regions. This was particularly evident for SIV integration sites, 174 of 328 (53%) of which occurred in regions of the genome whose gene density is higher than 11 genes per Mbp, compared to 149 of 432 (34%) and 17% for the MLV and the in silico, random sets, respectively. These data point out another difference between MLV- and SIV-derived vectors, the latter exhibiting a marked tendency to target gene-rich regions of the genome (*p* < 0.00001 using an omnibus contingency Chi^2^ test).

Recent studies have shown that about 30 highly gene dense clusters, called “ridges” (a loose acronym for “regions of increased gene expression”), are distributed among chromosomes. These ridges are characterized by typical expression levels per gene up to seven times higher than the genomic average (Caron et al. 2001). This feature is particularly evident for Chromosomes 3 and 6 (Versteeg et al. 2003). When looking at the distribution of retroviral integration sites on Chromosome 6 (Figure 3B), 22 out of the 30 SIV integration events (73%) fall within this unique ridge, a region of 20 Mbp (12% of Chromosome 6) with a density of 24 genes per Mbp, corresponding to the major histocompatibility complex region. This tendency to target gene-rich regions is less obvious for the MLV vector, which had only 7 out of 24 integration sites (29%) within this ridge (*p* < 0.005). Of the 22 SIV proviruses within this 20-Mbp ridge, ten were found clustered within a 2-Mbp, extremely gene-dense region (62 RefSeq genes per Mbp). Unexpectedly, only two out of these ten integration sites are inside transcription units, underscoring the strong tendency of SIV vectors to target gene-rich regions of the genome even if not within genes. Another feature of ridges is that they are noticeably enriched for short interspersed element (SINE), but depleted for long interspersed element (LINE) repeats (Versteeg et al. 2003). This correlation between SINE repeat density, GC content, and gene density has been previously reported (Bernardi et al. 1985) and may account for our observation of overrepresentation of integration events in SINE versus LINE elements, with 119 of 501 (24%) of the SIV set of integration sites being within SINE repeats.

### Common Integration Sites Differ between SIV- and MLV-Derived Vectors

Given their distinct patterns of integration, we compared identified common integration sites of MLV and SIV vectors. Using the definition of a common integration site as two or more proviruses integrated within a transcription unit (Suzuki et al. 2002), we have identified 40 genes targeted more than once by MLV and/or SIV vectors (Table S2). Of the RefSeq genes targeted by MLV and SIV vectors, 16 of 199 (8%) and 19 of 222 (9%), respectively, were hit at least twice, and ten genes were identified as common integration sites because they harbor both MLV and SIV proviruses. These genes have been targeted two times (*n* = 32), three times (*n* = 6), five times (*n* = 1), and seven times (*n* = 1). Among these 40 genes, seven are known to be involved in oncogenic translocations: *ARHGEF12, MDS1, MKL1, MSF, HMGA2, RAD51L1,* and *RUNX1*. Seven independent integration events have been identified in *MDS1*, predominantly within the second intron, 20–180 kb upstream of the first intron of *EVI1*.

## Discussion

A better understanding of retroviral integration patterns has evolved due to the availability of the complete murine and human genome sequences. Prior mapping studies have been performed in cell lines or in primary cells cultured short-term in vitro. However, integration site patterns may be cell type–dependent, for instance, if gene activity impacts integration site selection (Schroder et al. 2002), or if specific integrations facilitate engraftment and long-term contribution to hematopoiesis. Our aim was to provide a comprehensive comparative analysis of integration sites distribution of MLV- and SIV-derived vectors in long-term repopulating HSCs. Nonhuman primates have been shown to closely predict results in human transplantation and gene therapy clinical protocols (Donahue and Dunbar 2001) and thus represent the best currently available approach to generate information with relevance to design of future human clinical trials.

MLV-derived retroviruses are currently the most widely used vectors in clinical gene transfer protocols. Reports of proto-oncogene activation by replication-defective MLV vectors in mice and humans mandate more detailed evaluation of their potential for insertional mutagenesis. Separating the impact of overexpressing a growth-altering transgene from the insertional events themselves is particularly important to assess in primary repopulating HSCs. The main limitation of murine oncoretroviruses as gene therapy vectors is the requirement that cells pass through mitosis in order for the PIC to reach the nucleus and integrate. Since lentiviruses can transduce noncycling cells, lentivirus-based vectors have been actively developed and a clinical trial using these vectors has commenced. A detailed analysis of lentivector integration patterns is essential to assess the risk of insertional mutagenesis of these vectors compared to MLV vectors.

Although HIV-derived vectors can enter Old World monkey cells, they encounter a block prior to reverse transcription that is mediated by the dominant repressive factor TRIM5α, a component of cytoplasmic bodies (Stremlau et al. 2004), and thus are very inefficient at transducing nonhuman primate cells (An et al. 2000; Horn et al. 2002). Lentiviral vectors derived from SIV have been generated (Hanawa et al. 2004; Negre and Cosset 2002) and are useful for preclinical testing in nonhuman primates. SIV vectors may also be used to transduce human cells, and offer a number of potential advantages over HIV vectors for eventual clinical applications, such as lack of seroconversion to HIV positivity after exposure.

Only limited information exists regarding the rhesus monkey genome, but paleontological and genomic sequence data suggests that Macaca mulatta is 92.5%–95% identical to the humans at a DNA level (Page and Goodman 2001; Stewart and Disotell 1998). Moreover, the human and macaque karyotypes are virtually identical, with near absence of interchromosomal rearrangements and no detectable segments of nonhomology in euchromatic regions (Best et al. 1998; Muller and Wienberg 2001). We believe that this evolutionary information, combined with the characteristics of the sequences obtained in our study, validates our use of the human genome sequence to localize rhesus genomic insertion sites.

Analysis of SIV integration shows a striking tendency to integrate within transcription units (73% of the mapped integration events), but no propensity toward integration in any specific region of the transcription units, in contrast to MLV vectors. Although we did not observe regional hot spots for SIV integration, as previously reported in cell lines for HIV (Schroder et al. 2002), we instead noted the clustering of integrations within gene-rich regions. This penchant for integrating in so-called ridges may offer clues to a specific mechanism of integration. Loops of chromatin extending away from chromosome territories are frequently observed on the major histocompatibility complex locus of Chromosome 6, the ridge shown in Figure 3B, especially when transcription is induced (Mahy et al. 2002; Volpi et al. 2000). These data suggest that the formation of decondensed chromatin territories might be driven by transcription (Chubb and Bickmore 2003) to establish a nuclear environment accessible to transcription factors (Gilbert et al. 2004) and, therefore, to lentiviral PICs. This hypothesis is corroborated by the fact that genes targeted by SIV vectors tend to be more highly expressed in human CD34^+^Rho^lo^ cells, as compared to the total set of 33,000 expressed sequences analyzed on a standard expression array (Figure S1). Interestingly, functional analysis of genes identified as targets for SIV insertion using the Gene Ontology classification (Ashburner et al. 2000) and the EASE bioinformatics software (Hosack et al. 2003) shows a statistically significant overrepresentation of genes coding for transcription factors and nuclear proteins (Figure S2), suggesting either these genes are more concentrated in targeted areas of the genome or they share common genomic motifs or cellular proteins.

This striking tendency was not observed with the MLV-derived set of integration sites. While the ratio of MLV integration sites within transcription units was significantly higher than expected compared to in silico-generated random integration sites, the MLV proviruses displayed a unique and specific affinity for the region surrounding the transcription start site of annotated genes. The finding that among the 491 MLV integration sites, only 12% are within SINEs or LINEs may support the fact that MLV inserts into 5′ regulatory elements where insertions of transposable elements are probably strongly selected against. This also indicates tethering between some transcription factor(s) and MLV PIC protein(s).

These observations, consistent with previous comparative analyses in vitro (Wu et al. 2003; Mitchell et al. 2004), likely reflect the vectors' distinct mechanisms for accessing DNA and integrating, and may have implications for the relative risk of insertional mutagenesis. While replication-competent oncoretroviruses have been widely used to identify genes involved in cancer (Dudley 2003), insertional oncogenesis has, to our knowledge, never been clearly reported after lentiviral infection. Both vectors have drawbacks: MLV vector integrations near the 5′ ends of genes may be more likely to disrupt transcriptional control and result in dysregulated expression of potentially oncogenic gene products, while SIV vector insertions within transcription units might be more likely to result in frame shifts or other events abrogating production of the normal gene product. Thus, the possibility that SIV vectors are less likely than MLV vectors to induce tumorigenesis needs to be carefully evaluated in relevant animal models.

A large number of genes were identified with two or more integration events, and thus were deemed common integration sites, including ten genes that had both MLV and SIV integrations. This suggests either that these genes are particularly susceptible to integration events due to open chromatin or other factors that favor both types of viruses, or that integration events in these particular genes alter expression and favor engraftment and long-term contributions to hematopoiesis. However, the most striking finding was the occurrence of seven independent hits by MLV in the first two introns of the *MDS1* gene, whereas *MDS1* was not found in the SIV dataset of integration sites. *MDS1* is adjacent to the *EVI1* locus, which has been implicated as a retrovirally activated proto-oncogene in a number of murine leukemogenesis studies (Bartholomew et al. 1989; Bordereaux et al. 1987; Morishita et al. 1988; Li et al. 2002). This unexpected and highly nonrandom clustering raises several questions since recent mapping analyses in cell lines did not report any common integration site (Wu et al. 2003). This suggest that proviral insertion near a proto-oncogene *(MDS1/EVI1)* may occur at a much higher frequency than previously expected. Studies are ongoing to better understand the causes and consequences of retroviral integration within this genomic locus.

It is important to stress that the very long-term follow-up of a large cohort of nonhuman primates, including all animals in the current study, has revealed completely normal hematopoiesis and lack of any progression towards neoplasia (Kiem et al. 2004). All animals have stable polyclonal hematopoiesis from transduced cells without any progression toward oligoclonality. Despite the nonrandom nature of integration and the possible targeting of certain proto-oncogenes, the use of replication-defective MLV or SIV vectors expressing nontransforming transgenes in the setting of one or very few integrants per cell still likely carries a very low risk of oncogenesis (Baum et al. 2003). Design of safer vectors including insulating elements to decrease the risk of activation of adjacent genes, development of targeted integration systems, or use of novel vectors with different integration patterns, should allow continued progress toward safe and effective gene therapy. However, for serious disorders such as SCID, even current MLV vectors are likely justified.

## Materials and Methods

### 

#### Rhesus macaque autologous transplantation model

Rhesus macaques were handled in accordance with the guidelines set by the Committee on Care and Use of Laboratory Animals of the Institute of Laboratory Animal Resources (National Research Council 1985). Protocols were approved by the Animal Care and Use Committee of the National Heart, Lung, and Blood Institute. Details of mobilization, transduction, and transplantation were previously published (Hanawa et al. 2004; Hematti et al. 2003; Takatoku et al. 2001; Wu et al. 2000). Animals were mobilized with stem cell factor (SCF) and granulocyte colony-stimulating factor for five doses, and underwent apheresis on day 5. CD34^+^ cells were enriched from mobilized PB by immunoabsorption and transduced for 96 h with either amphotropic MLV vectors LNL6 and G1Na containing the neomycin resistance gene (*n* = 22) (Miller and Buttimore 1986), or for 48 h with amphotropic SIV vector containing the *green fluorescent protein* gene (*n* = 3) (Hanawa et al. 2004). All transduction cultures were carried out in the presence of 100 ng/ml Flt3 ligand, 100 ng/ml SCF, and either 20 ng/ml interleukin-3 and 50 ng/ml interleukin-6 (*n* = 12), or 100 ng/ml megakaryocyte growth and development factor (*n* = 10 MLV animals and all SIV animals). All animals received cells transduced on flasks coated with Retronectin (TaKara, Shiga, Japan). In addition, two MLV animals also received cells transduced on autologous marrow stromal cells (Wu et al. 2000). Cells were reinfused intravenously following 1,000 rads of total body irradiation. PB samples were collected at a minimum of 6 mo after transplantation from three animals receiving SIV-transduced cells and 22 receiving MLV-transduced cells. MNCs were isolated by density gradient centrifugation over lymphocyte separation medium (Organon Teknika, Durham, North Carolina, United States), and granulocytes were obtained as previously described (Tisdale et al. 1998).

#### Cloning of the integration sites by LAM-PCR

LAM-PCR and cloning of insertion site vector genomic fusion sequences was performed as described (Hanawa et al. 2004; Schmidt et al. 2002) using 5′-linker cassettes and 3′-LTR primers designed specifically for MLV- or SIV-based vectors (Hanawa et al. 2004) (Table S3). Amplicons of junctions between genomic regions and 5′-LTRs were purified from agarose gels and cloned with the TOPO TA cloning kit (Invitrogen, Carlsbad, California, United States). Cycle sequencing was performed using an ABI Prism Genetic Analyzer 3100 (Applied Biosystems, Foster City, California, United States). Sequences were analyzed using Lasergene software (Dnastar, Madison, Wisconsin, United States).

#### Creation of a control set of in silico-generated integration sites

For statistical comparison to the integration site sets, we computationally generated 1,000 sets of integration sites. For MLV, we made 1,000 datasets, each containing 432 randomly selected genomic coordinates; for SIV, we made 1,000 datasets of 328 points each. All human chromosome sequences were concatenated into a single long sequence. We used the random number generator function in Perl to pick a number between 1 and the total number of nucleotides in the human genome (3,098,026,039), then identified this position in the concatenated sequence and correlated this position back to its chromosomal origin. If this coordinate fell within a sequencing gap, a new number was picked. We performed an ANOVA on the in silico-generated integration sites to demonstrate that 1,000 random sets were sufficient (unpublished data).

#### Genomic analysis of the retroviral and in silico-generated integration sites

We used a bioinformatic pipeline (Crawford et al. 2004) to map the position of each retroviral and in silico-generated integration site relative to 20,623 National Center for Biotechnology Information mRNA RefSeqs aligned by the UCSC Genome Browser. For each integration site, we calculated the distance to the nearest 5′ and 3′ end of a RefSeq gene. We disregarded cases in which RefSeq mRNAs aligned only partially to the genome. Genomic location of all LTR coordinates are available through the UCSC Genome Browser Custom Tracks (available at http://research.nhgri.nih.gov/projects/Dunbar/May2004/). Two-sided *p* values were obtained using the Chi^2^ test.

#### Functional clustering and over-representation analysis of targeted genes

Genes identified as targeted by retroviral insertion were analyzed for significant functional clusters of genes using the EASE bioinformatics software (http://david.niaid.nih.gov/david/ease.htm). This software was used to rank functional clusters by statistical overrepresentation of individual genes in specific categories relative to all genes in the same category. The functional clusters used by EASE were derived from the Gene Ontology classification system (http://www.geneontology.org).

## Supporting Information

Dataset S1 MLV-Derived Integration Site Sequences (FASTA Format) (78 KB TXT).

Dataset S2 SIV-Derived Integration Site Sequences (FASTA Format) (60 KB TXT).

Figure S1 Relative Expression of Genes with Identified MLV and SIV Integrations, Using Data from Human CD34^+^Rho^lo^ Cells (38 KB PDF).

Figure S2 Gene Ontology Categories Statistically Overrepresented in the Genes Targeted by SIV-Derived Vector (16 KB PDF).

Table S1 Comparison of Retroviral Integration Sites Distribution within Transcription Units (12 KB PDF).

Table S2 RefSeq Genes Targeted More than Once by SIV, MLV, or Both Retroviral Vectors (18 KB PDF).

Table S3 Primers Used for the LAM-PCR Experiments (8 KB PDF).

### Accession Numbers

The retroviral integration site sequences larger than 50 bp discussed in this paper have been deposited in GenBank (http://www.ncbi.nlm.nih.gov/Genbank/) under the accession numbers AY728482 to AY728804 for SIV, and AY733679 to AY734083 for MLV.

LocusLink ID numbers (http://www.ncbi.nlm.nih.gov/LocusLink/) for the genes discussed in this paper are *ARHGEF12* (23365), *EVI1* (2122), *HMGA2* (8091), *LMO2* (4005), *MDS1* (4197), *MKL1* (57591), *MSF* (10801), *RAD51L1* (5890), *RUNX1* (861), and*TRIM5α* (85363).

## Abbreviations

HSC: hematopoietic stem cell
LAM-PCR: linear amplification-mediated PCR
LINE: long interspersed element
LTR: long terminal repeat
MLV: murine leukemia virus
MNC: mononuclear cell
PB: peripheral blood
PIC: preintegration complex
RefSeq: reference sequence
SCID: severe combined immunodeficiency
SINE: short interspersed element
SIV: simian immunodeficiency virus
UCSC: University of California
