# Supplementary material for: Distinct Genomic Integration of MLV and SIV Vectors in Primate Hematopoietic Stem and Progenitor Cells
Source: PLoS Biol. 2004 Nov 23;2(12):e423. doi: 10.1371/journal.pbio.0020423 (PMC529319; doi:10.1371/journal.pbio.0020423)
Supplement: Figure S1 — (38 KB PDF). [file pbio.0020423.sg001.pdf]

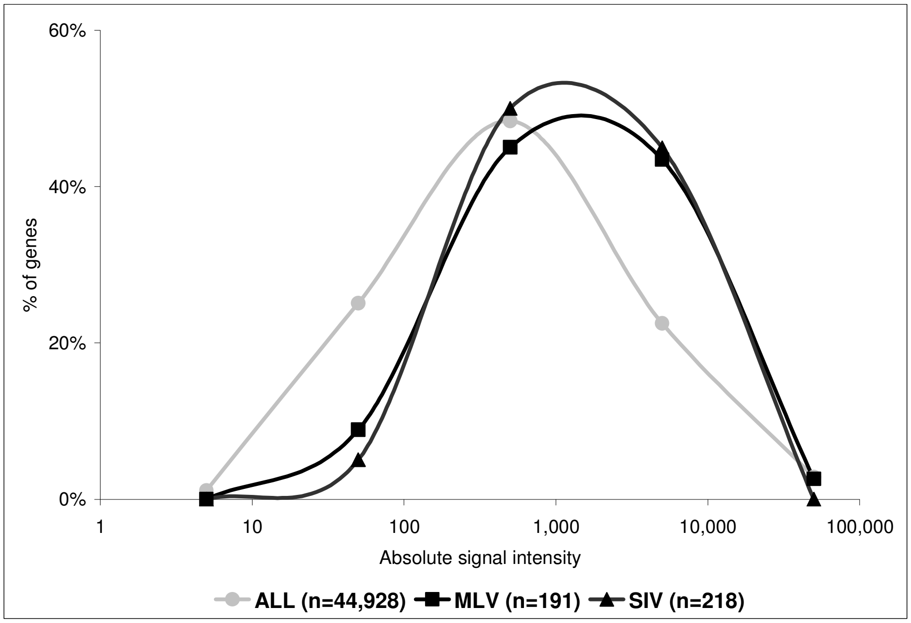

Figure S1. Relative expression of genes with identified MLV and SIV integrations compared to all genes, using data from human PB-mobilized $CD34^{+}Rho^{lo}$ cells.

Expression experiments using RNA from rhesus cells and human microarrays transcripts are difficult to interpret since single nucleotide changes in transcripts may result in partial hybridization. Consequently, we have used the Affymetrix Human Genome U133 Set GeneChip array, that contains 44,928 probe sets representing 33,000 well-substantiated human genes, to analyze the expression pattern of retrovirally-targeted genes in human PB-mobilized $CD34^{+}Rho^{lo}$ cells, the best approximation of a human or primate stem cell population likely closely reflecting the cell population originally transduced with MLV or SIV in these animals. 96% and 98% of the genes targeted by MLV and SIV vectors were present on the chips, respectively. The median expression level of the sets of genes identified as MLV or SIV integration sites were compared to the overall median expression level of all genes in the $CD34^{+}Rho^{lo}$ cell population. The sets of genes targeted by the SIV-derived proviruses have significantly more genes with high median expression levels than all genes represented on the microarrays ($p<6.9e-6$). Genes hosting MLV proviruses display a lower median expression level, with a less marked but still statistically significant increase over the entire gene set ($p<0.033$). These findings

are similar to recent *in vitro* datasets (Mitchell et al. 2004), and probably reflect different chromosomal influences on integration targeting by MLV and SIV-derived vectors.

Materials and Methods

Isolation of $CD34^+Rho^{lo}$ cell population from mobilized PB. Mobilized PB from healthy donors was obtained after informed consent in accordance with guidelines approved by the University of Minnesota Committee on the Use of Human Subjects in Research. Each replicate was comprised of an individual donor for mobilized PB samples. MNCs were isolated by density gradient centrifugation using Histopaque®-1077 (Sigma-Aldrich Co., Saint Louis, MO, USA). The $CD34^+$ fraction of the MNCs was enriched to >95% purity by two consecutive rounds of magnetic-activated cell sorting (MACS) using the Direct CD34 Progenitor Isolation Kit (Miltenyi Biotec, Auburn, CA, USA). $CD34^+$ enriched cells were resuspended in Iscove's Medium (Mediatech, Herndon, VA, USA) supplemented with 10% fetal bovine serum (FBS) (HyClone, Logan, UT, USA) and 150 ng/mL Rhodamine 123 (Rho) (Sigma-Aldrich Co.), incubated at 37°C for 30 min, washed and incubated for an additional 30 min at 37°C in Iscove's Medium + 10% FBS. Subsequently, cells were stained with allophycocyanin (APC)-conjugated anti-CD38, APC-conjugated anti-CD33 and phycoerythrin (PE)-conjugated anti-c-kit monoclonal antibodies or the appropriate isotype control antibodies (all from BD Biosciences, San Jose, CA, USA) on ice for 30 min. The $CD34^+CD38^-CD33^-$c-kit^+Rho^{lo} ($CD34^+Rho^{lo}$) and $CD34^+CD38^-CD33^-Rho^{hi}$ fraction of each sample was isolated by fluorescence-activated cell sorting (FACS) using a FACSVantage SE system with the DiVa upgrade (BD Biosciences) at the University of Minnesota Cancer Center Flow Cytometry Core Facility.

Isolation of total RNA, linear amplification and labeling for microarray analysis. Total cellular RNA was isolated from mobilized PB (n=3) $CD34^+Rho^{lo}$ cells using the PicoPure RNA Isolation Kit (Arcturus, Mountain View, CA, USA). Seven to 10 thousand $CD34^+Rho^{lo}$ cells were sorted directly into 100µL Extraction Buffer provided with the PicoPure RNA Isolation Kit (Arctutus). Labeled complimentary-RNA (cRNA) for Affymetrix™ gene chip hybridization was generated by one round of IVT-based, linear amplification using the RiboAmp OA RNA Amplification Kit (Arcturus) followed by

labeling with the Enzo Bioarray™ HighYield™ RNA Transcript Labeling Kit (Enzo Life Sciences, Farmingdale, NY, USA) according to manufacturer's instructions.

Gene-expression profiling. The Affymetrix standard protocol was used to prepare triplicate samples with 5µg of $CD34^{+}Rho^{lo}$ total RNA for hybridization to the Human Genome U133 GeneChips, containing approximately 45,000 probe sets representing 33,000 well-substantiated human genes. The median intensity of all chips were scaled to a value of 500 using global scaling in the Affymetrix Microarray Suite software (Version 5.0). The data from the separate arrays were combined by scaling the B chip expression data so that the to the mean expression value of the probeIDs common to the A and B arrays was the same. In order to determine the statistical significance of the RefSeqs identified using the bioinformatics pipeline, we determined which probesets on the U133 array had an expression level greater than the mean. Using this probeset list as a basis, we determined whether the subset of genes defined in the RefSeq list represented genes that have expression levels deviating from the overall expression pattern in a statistically significant manner using a hypergeometric distribution (without a multiple testing correction).
