# Supplementary material for: Distinct Genomic Integration of MLV and SIV Vectors in Primate Hematopoietic Stem and Progenitor Cells
Source: PLoS Biol. 2004 Nov 23;2(12):e423. doi: 10.1371/journal.pbio.0020423 (PMC529319; doi:10.1371/journal.pbio.0020423)
Supplement: Figure S2 — (16 KB PDF). [file pbio.0020423.sg002.pdf]

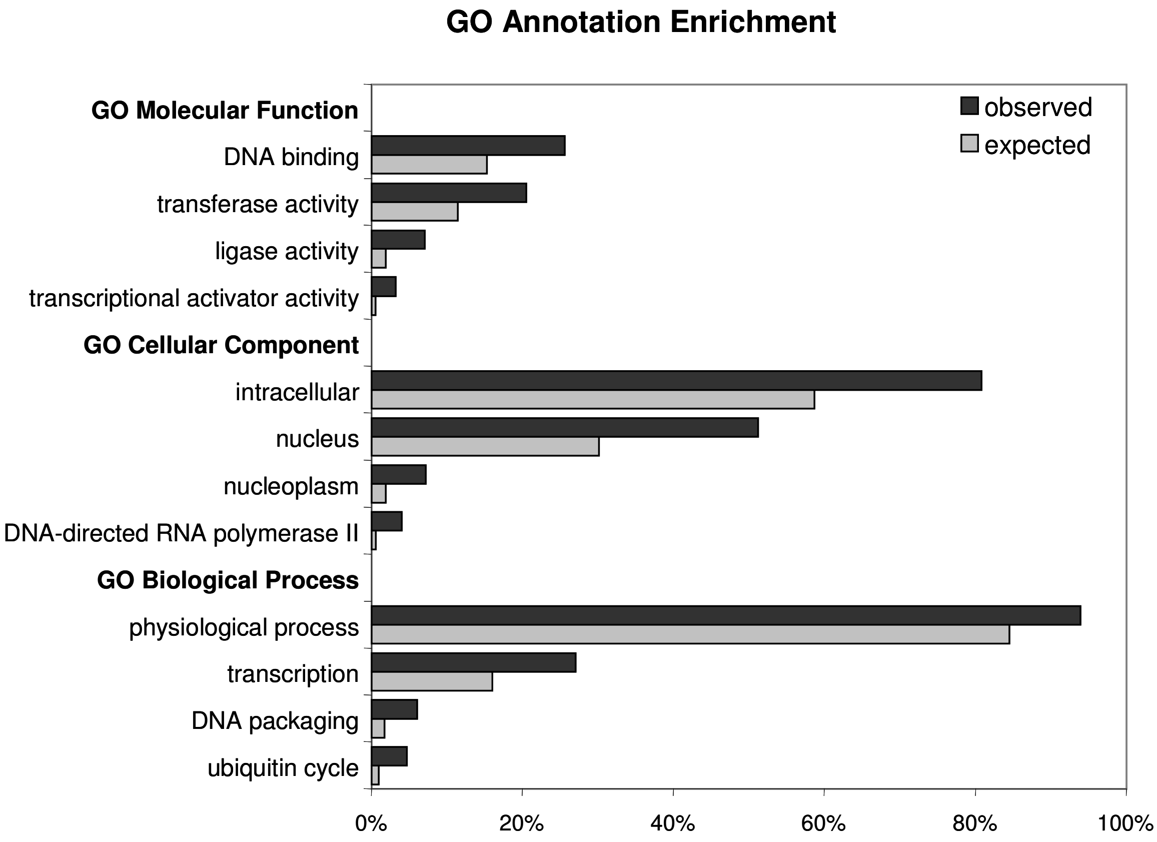

Figure S2. Gene Ontology categories statistically over-represented in the genes targeted by SIV-derived vector. Genes identified as targets for proviral insertion were analyzed for significant functional clusters of genes with the EASE bioinformatics software package. The functional clusters used by EASE were derived from the Gene Ontology classification. This program was used to rank functional clusters by statistical over-representation of individual genes in specific Gene Categories relative to all GO-annotated genes in the same category. Dark grey bars ("observed") is the percentage of SIV-targeted genes in each Category relative to the total number of SIV-targeted genes annotated within the given Gene Category; the light grey bars ("expected") is the percentage of gene identifiers (GeneID) that belong to the specific Gene Category relative to the number of gene identifiers annotated within the given Gene Category. The p value of the one-tailed Fisher exact probability of over-representation is <0.0001 for all the represented categories.
