# Supplementary material for: Distinct Genomic Integration of MLV and SIV Vectors in Primate Hematopoietic Stem and Progenitor Cells
Source: PLoS Biol. 2004 Nov 23;2(12):e423. doi: 10.1371/journal.pbio.0020423 (PMC529319; doi:10.1371/journal.pbio.0020423)
Supplement: Table S1 — (12 KB PDF). [file pbio.0020423.st001.pdf]

	MLV (n=432)	SIV (n=328)	MLV (n=459)	SIV (n=362)
Identity to the human genome	at least 90%		at least 80%	
Inside RefSeq genes	49.1%	73.5%	49.7%	72.9%

Table S1. Comparison of retroviral integration sites distribution within transcription units with and without the rhesus sequences that were less than 90% orthologous to the human genome.
