# Supplementary material for: Distinct Genomic Integration of MLV and SIV Vectors in Primate Hematopoietic Stem and Progenitor Cells
Source: PLoS Biol. 2004 Nov 23;2(12):e423. doi: 10.1371/journal.pbio.0020423 (PMC529319; doi:10.1371/journal.pbio.0020423)
Supplement: Table S2 — (18 KB PDF). [file pbio.0020423.st002.pdf]

	# hits	Chr.	Gene size (bps)	Gene Symbol	Gene ID	Gene Name
SIV	2	1	155,979	MACF1	23499	microtubule-actin crosslinking factor 1
	3	1	114,312	EYA3	2140	eyes absent homolog 3
	2	1	92,591	SHARP	23013	SMART/HDAC1 associated repressor protein
	2	2	168,390	TLK1	9874	tousled-like kinase 1
	2	3	55,957	SACM1L	22908	SAC1 suppressor of actin mutations 1-like (yeast)
	2	3	103,954	PIK3CB	5291	phosphoinositide-3-kinase, catalytic, beta polypeptide
	2	6	53,577	STK38	11329	serine/threonine kinase 38
	2	9	207,638	VPS13A	23230	vacuolar protein sorting 13A
	3	11	152,699	ARHGEF12	23365	Rho guanine nucleotide exchange factor 12
	2	16	50,434	ITGAL	3683	integrin, alpha L
	2	16	141,462	NFATC3	4775	nuclear factor of activated T-cells, cytoplasmic, calcineurin-dependent 3
	2	17	73,088	PRPSAP2	5636	phosphoribosyl pyrophosphate synthetase-associated protein 2
	2	17	32,431	182-FIP	57532	82-kD FMRP interacting protein
	3	17	296,747	SCAP1	8631	src family associated phosphoprotein 1
	2	17	83,060	APPBP2	10513	amyloid beta precursor protein binding protein 2
	2	17	30,067	PCNT1	79902	pericentrin 1
	2	20	27,021	C20orf140	128637	chromosome 20 open reading frame 140
	2	22	226,355	MKL1	57591	megakaryoblastic leukemia 1
MLV	2	1	46,142	FLJ32112	127428	hypothetical protein FLJ32112
	2	2	80,791	LPIN1	23175	lipin 1
	2	2	45,728	CASP10	843	caspase 10
	2	3	136,110	TRAD	11139	serine/threonine kinase with Dbl- and pleckstrin homology domains
	7	3	514,588	MDS1	4197	myelodysplasia syndrome 1
	3	8	186,230	RBPMS	11030	RNA binding protein with multiple splicing
	2	14	699,786	IGH	3492	immunoglobulin heavy locus
	2	16	23,900	LOC57149	57149	hypothetical protein A-211C6.1
	2	17	180,525	MSF	10801	MLL septin-like fusion
	2	18	140,154	C18orf17	125488	hypothetical protein FLJ33761
	2	18	88,720	PSTPIP2	9050	proline-serine-threonine phosphatase interacting protein 2
	2	20	67,976	RRBP1	6238	ribosome binding protein 1 homolog
MLV and SIV	2	4	59,726	MMRN	22915	multimerin
	2	4	402,638	INPP4B	8821	inositol polyphosphate-4-phosphatase
	2	6	462,343	ATXN1	6310	spinocerebellar ataxia 1
	2	9	40,094	TRAF2	7186	TNF receptor-associated factor 2
	5	11	174,389	PACS1	55690	phosphofurin acidic cluster sorting protein 1
	2	12	141,808	HMGA2	8091	high mobility group AT-hook 2
	2	14	678,633	RAD51L1	5890	RAD51-like 1
	3	17	87,576	GRB2	2885	growth factor receptor-bound protein 2
	2	19	15,395	ZNF432	9668	zinc finger protein 432
	3	21	261,536	RUNX1	861	runt-related transcription factor 1

Table S2. List of RefSeq genes targeted more than once by SIV, MLV or both retroviral vectors. The number of hits within each transcription unit is listed in the first column and the chromosomal location in the second column.
