# Supplementary material for: Distinct Genomic Integration of MLV and SIV Vectors in Primate Hematopoietic Stem and Progenitor Cells
Source: PLoS Biol. 2004 Nov 23;2(12):e423. doi: 10.1371/journal.pbio.0020423 (PMC529319; doi:10.1371/journal.pbio.0020423)
Supplement: Table S3 — (8 KB PDF). [file pbio.0020423.st003.pdf]

Linear PCR	LTR-b	GGCAGGAACTGCTTACCA
1st PCR	LTR-R1	CAGCTGTTCCATCTGTTC
	LC1	GACCCGGGAGATCTGAAT
Nested PCR	LTR-R2	GCTAGCTTGCCAAACCTAC
	LC2	CAGTGGCACAGCAGTTAGGA

Table S3. List of primers used for the LAM-PCR experiments.
